# Analysis of cross-reactivity between radio -contrast media in 97 hypersensitivity reactions

**DOI:** 10.1186/2045-7022-4-S3-P133

**Published:** 2014-07-18

**Authors:** Berangere Lerondeau, Philippe Trechot, Christophe Paris, Amandine Luc, Julie Waton, Claire Poreaux, Jean-Luc Schmutz, Annick Barbaud

**Affiliations:** 1 University Hospital of Nancy, Dermatology and Allergology Department, France; 2 University Hospital of Nancy, Pharmacology Department, France; 3 University Hospital of Nancy, Research Unit EA 72-98 “INGRES”, France; 4 Lorraine University, Research Unit EA 72-98 “INGRES”, France

## Background

Sensitized skin tests can be used to analyze cross-reactivity (CR) between radio -contrast media (RCM) and to guide the choice of alternative RCM for subsequent injections.

## Objective

To analyze the CR between different RCM.

## Methods

Between 2006 and 2012, a retrospective study from was performed using data collected from patients who had skin tests with nine different RCM after a non-tolerated injection of RCM, followed by a substitution test with another RCM. Patients with a positive test patch, prick or intradermal test and/or a non-tolerated re-challenge of RCM were included.

## Results

Ninety-seven patients were included. CR occurred between ionic monomers (IMs) in 2 cases, between non-ionic monomers (NIMs) in 93 cases, between an ionic dimer (ID) and an IM in 1 case, between NIMs and IMs in 38 cases, between NIMs and IDs in 29 cases, between non-ionic dimers (NIDs) and IMs in 9 cases, between IDs in 13 cases, and between NIMs in 65 cases. Multiple correspondence analyses identified two subgroups of RCM in which CR was frequent: Group A (iodixanol, iopamidol, iomeprol, iohexol, ioversol), which share two identical N-(2, 3-dihydroxypropyl) carbamoyl side chains that could be the antigen determinant (also present in other drugs), and Group B (ioxaglate, iobitridol).

## Conclusion

From this largest study of CR between RCM, we demonstrate that frequent CR between RCM does not follow the current chemical classification. Thus, we propose reclassifying RCM, and also suggest re-challenging sensitized patients with RCM from a different CR subgroup than that of the RCM initially involved.

